# Eczema mpoxicum: Extensive secondary mpox infection in a man with severe atopic dermatitis and HIV

**DOI:** 10.1016/j.jdcr.2026.05.022

**Published:** 2026-05-15

**Authors:** Ameya Gangal, Haya Raef, Benjamin K. Stoff, Justin Cheeley

**Affiliations:** Department of Dermatology, Emory University School of Medicine, Atlanta, Georgia

**Keywords:** atopic dermatitis, eczema, HIV, infection, mpox, skin of color, vaccination

Mpox, a zoonotic double-stranded DNA virus of the *Orthopoxvirus* genus, classically presents with a prodrome of fever, lymphadenopathy, and culminates in facial lesions resembling smallpox.^1^ Following the May 2022 reports of infection in nonendemic areas, mpox has been reported several times with limited lesion counts (<50) involving the genitals, face, or extremities particularly among patients with human immunodeficiency virus (HIV) and gay or bisexual men with recent unprotected sexual encounters.^1^ Barring a report of 80 mpox lesions in a patient with atopic dermatitis (AD), there are no reports of extensive secondary infection of AD.^2^ Here, we present a case of extensive secondary infection of mpox on AD, termed eczema mpocixum.

## Case report

A 26-year-old man with history of severe AD and HIV diagnosed 5 months prior to presentation, on antiretroviral therapy with undetectable viral load and CD4 count of 876 (26.8%), presented with a 4-day history of a mildly pruritic, nontender eruption. Of note, the patient’s severe AD was previously inadequately controlled despite interleukin (IL)-4/13 inhibition. Two years prior to evaluation, he was transitioned to upadicitinib monotherapy complicated by several episodes of eczema herpeticum. Upadicitinib was discontinued 3 months prior to evaluation, at which time he was not on systemic therapy for AD.

**Fig 1:**
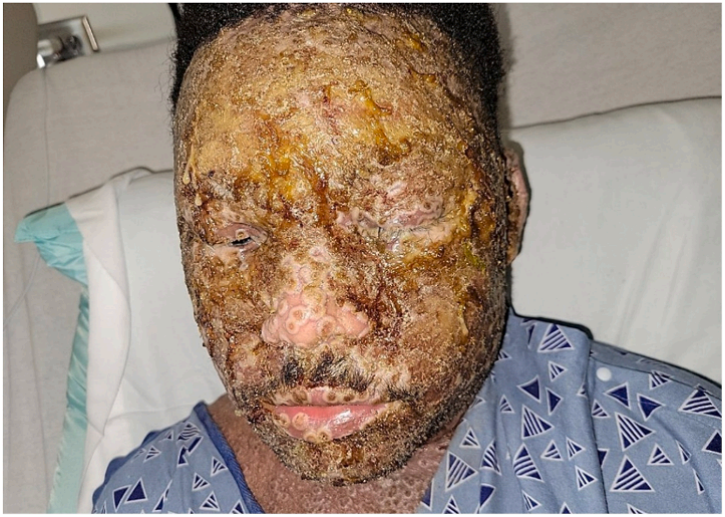
Confluent yellow crust and prominent facial edema with discrete and coalescing heaped-up gray ovoid umbilicated papulovesicles.

**Fig 2:**
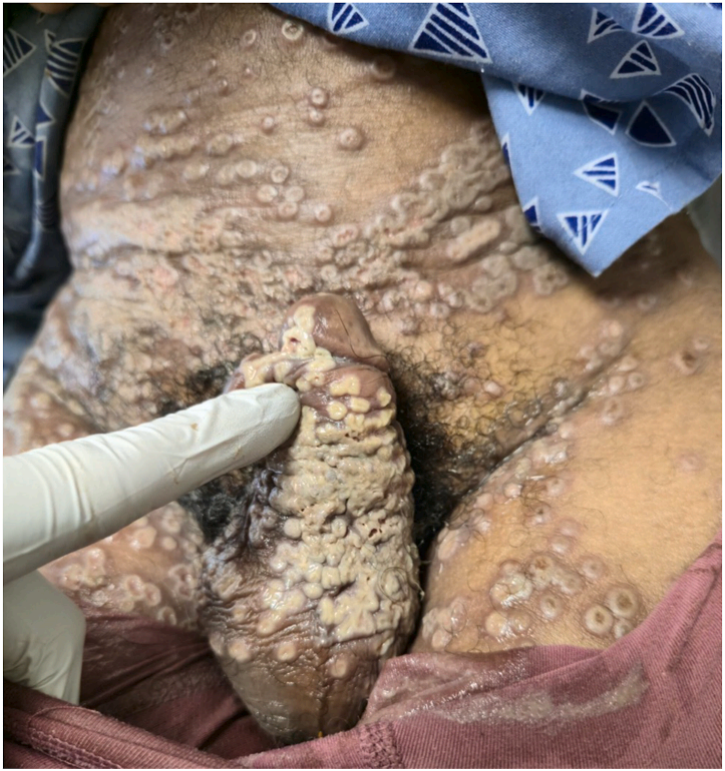
Yellow-gray umbilicated papulovesicles coalescing into a plaque along the ventral penile shaft with innumerable heaped-up papulovesicles noted on the abdomen and thighs.

**Fig 3:**
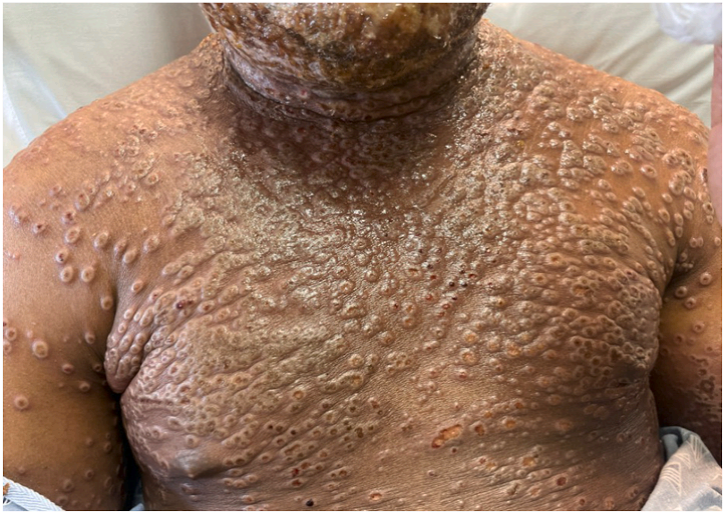
Innumerable heaped-up umbilicated papulovesicles along the chest and neck.

The exanthem initially involved the lips and genitals, then spread to the face, trunk, and extremities. He was afebrile and tachycardic with a pulse between 100 and 120 beats per minute. Physical examination revealed innumerable, heaped-up, gray papulovesicles with central umbilication involving approximately 80% of the body surface area. Prominent facial edema and crusting was also noted, without appreciable cervical, axillary, or inguinal lymphadenopathy. He reported unprotected anal-oral, penile-oral, and anal receptive intercourse with a male partner 10 days prior to presentation (Fig 1, Fig 2, Fig 3).

**Fig 4:**
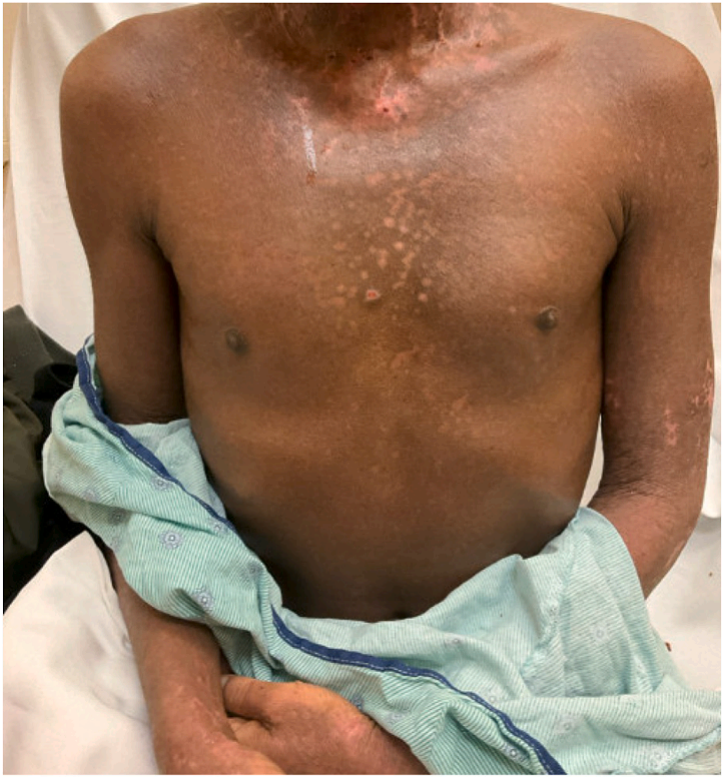
Varioliform scarring and postinflammatory hypopigmentation at sites of most prominent mpox involvement along the chest 2 months after discharge.

Deroofed lesional fluid was positive for mpox virus on gene amplification assays from two distinct samples collected on hospital day 0 and hospital day 2. Herpes simplex virus polymerase chain reaction assays were negative from 2 deroofed lesional fluid samples. A skin biopsy of the right arm demonstrated epidermal ulceration with extensive necrosis and focal features of viral cytopathic changes, including multinucleated cells within keratinocytes. Ophthalmology consultation revealed a 1 ×2 mm corneal abrasion without vitritis, disc edema, or dendritic staining. Rapid plasma regain was nonreactive. Bacterial culture on admission grew methicillin sensitive *Staphylococcous aureus* and group A β-hemolytic *Streptococcus*. Isolation measures were initiated in conjunction with gentle debridement, nonadhesive dressings, and skin protectants. Off-label therapies used for mpox throughout his hospitalization included topical cidofovir, oral tecovirimat, varicella immunoglobulin, and intravenous brincodofovir. Upadicitinib or other biologic therapies targeting his severe AD were not reinitiated during his hospitalization. Secondary bacterial infection was managed with linezolid and ertapenem. The patient’s hospital course was complicated by persistent tachycardia, pain, and frequent wound care, necessitating transfer to the intensive care unit. On hospital day 29, the patient was discharged home without longstanding complications (Fig 4).

## Discussion

We posit that a chronically damaged skin barrier, previous immunosuppression with oral Janus kinase inhibitor therapy, and HIV may have contributed to the extensive secondary mpox infection. Treatment of mpox is largely supportive, and there are no Food and Drug Administration-approved antiviral therapies.^3^

The described case underscores the importance of testing for uncommon sources of secondary infection in patients with severe AD when faced with a high degree of clinical suspicion based on lesion morphology. Thorough sexual history gathering further complements clinical examination findings suggestive of mpox. Male patients with severe AD who have sex with men should be encouraged to receive the JYNNEOS vaccine, a replication-deficient vaccinia virus without potential to produce secondary infection.^1^^,^^4^ Finally, multidisciplinary hospital care is of critical importance to attenuate viral shedding, limit secondary infection, control pain, and optimize skin healing, particularly among immunocompromised individuals at risk of severe systemic complications.

## Conflicts of interest

None disclosed
